# Assessing the robustness of COVID-19 vaccine efficacy trials: systematic review and meta-analysis, January 2023

**DOI:** 10.2807/1560-7917.ES.2023.28.22.2200706

**Published:** 2023-06-01

**Authors:** Thi Ngoc Anh Hoang, Ha-Linh Quach, Van Ngoc Hoang, Van Thien Tran, Quang Thai Pham, Florian Vogt

**Affiliations:** 1 Faculty of Medicine, PHENIKAA University, Yen Nghia, Ha Dong, Hanoi, Vietnam; 2 National Centre for Epidemiology and Population Health, Research School of Population Health, College of Health and Medicine, Australian National University, Canberra, ACT, Australia; 3 Department of Communicable Diseases Control and Prevention, National Institute of Hygiene and Epidemiology, Hanoi, Vietnam; 4 Centre for Ageing Research and Education (CARE), Duke-NUS Medical School, Singapore, Singapore; 5 The General Department of Preventive Medicine, Ministry of Health, Hanoi, Vietnam; 6 Hospital of Vietnam National University, Hanoi, Vietnam; 7 School of Preventive Medicine and Public Health, Hanoi Medical University, Hanoi, Vietnam; 8 The Kirby Institute, University of New South Wales, Sydney, New South Wales, Australia

**Keywords:** fragility index, fragility quotient, robustness, randomized controlled trial, COVID-19, SARS-CoV-2, vaccine efficacy

## Abstract

**Background:**

Vaccines play a crucial role in the response to COVID-19 and their efficacy is thus of great importance.

**Aim:**

To assess the robustness of COVID-19 vaccine efficacy (VE) trial results using the fragility index (FI) and fragility quotient (FQ) methodology.

**Methods:**

We conducted a Cochrane and PRISMA-compliant systematic review and meta-analysis of COVID-19 VE trials published worldwide until 22 January 2023. We calculated the FI and FQ for all included studies and assessed their associations with selected trial characteristics using Wilcoxon rank sum tests and Kruskal–Wallis H tests. Spearman correlation coefficients and scatter plots were used to quantify the strength of correlation of FIs and FQs with trial characteristics.

**Results:**

Of 6,032 screened records, we included 40 trials with 54 primary outcomes, comprising 909,404 participants with a median sample size per outcome of 13,993 (interquartile range (IQR): 8,534–25,519). The median FI and FQ was 62 (IQR: 22–123) and 0.50% (IQR: 0.24–0.92), respectively. FIs were positively associated with sample size (p < 0.001), and FQs were positively associated with type of blinding (p = 0.023). The Spearman correlation coefficient for FI with sample size was moderately strong (0.607), and weakly positive for FI and FQ with VE (0.138 and 0.161, respectively).

**Conclusions:**

This was the largest study on trial robustness to date. Robustness of COVID-19 VE trials increased with sample size and varied considerably across several other important trial characteristics. The FI and FQ are valuable complementary parameters for the interpretation of trial results and should be reported alongside established trial outcome measures.

**Key public health message**

**What did you want to address in this study?**

‘Trial robustness’ is an important yet underutilised concept to interpret results from randomised controlled trials (RCT). While good efficacy profiles have been reported for several COVID-19 vaccines, the robustness of the underlying trial results has not yet been established. We aimed to investigate the robustness of COVID-19 vaccine efficacy trials, through a synthesis of all published studies worldwide.

**What have we learnt from this study?**

We found that trial robustness was high overall, however, several important trial characteristics varied substantially across studies such as the number of trial participants, the method of blinding to minimise biases, the proportion of participants lost to follow-up, and the vaccine efficacy. Rigorous methodological standards for trial conduct are crucial to ensure high robustness of results.

**What are the implications of your findings for public health?**

COVID-19 vaccine trial results are overall very trustworthy, which is an important finding in times of rising vaccine hesitancy and scepticism towards medical research methods. Only reporting effect sizes and p-value-based ‘statistical significance’ does not fully capture the complexities of trial results. We recommend making standard practice the reporting of trial robustness for vaccine efficacy RCTs alongside established trial outcome measures.

## Introduction

Several vaccines against severe acute respiratory syndrome coronavirus 2 (SARS-CoV-2) have been and are being developed, and the scientific evidence about their efficacy is growing rapidly [1]. There are now several COVID-19 vaccines administered at global scale for which good efficacy profiles have been reported [2-4]. However, no solid evidence exists to date about the robustness of COVID-19 vaccines trial results. Given how crucial the vaccines have become in the global mitigation of COVID-19, this constitutes a major evidence gap.

Robustness of results from randomised controlled trials (RCT), or lack thereof, has been long recognised as an important issue in medical research [5,6]. Undue emphasis is often put on p values, often set at an alpha error level of 0.05 when interpreting trial results [7-9], though it is known that statistical significance in trials can easily be lost by small changes of only a few events in the intervention or control group [10]. In response to this, the concept of ‘trial fragility’ was developed by Feinstein and by Walter in the 1990s, as a way to quantify how robust, or fragile, results from RCTs are [11,12], but it took until 2014, when the concept of ‘fragility index’ (FI) was defined by Walsh et al., for FI to be used more frequently [13]. In short, the FI constitutes the minimum number of trial participants whose outcome event would need to change for the statistical significance of the entire trial to switch from ‘statistically significant’ to ‘statistically not significant’, with smaller FI indicating more fragile results; details and example calculation for the FI are presented in Supplement File 1 [13]. The FI does not aim to replace or invalidate the use of p values, but rather to provide a complementary, easily interpretable metric to gauge how robust a trial result is [14]. In the opposite case of statistically non-significant results, the reverse fragility index (RFI) can be calculated instead [15]. In contrast to the FI, the RFI represents the minimum number of events needed to reverse a non-significant result to a significant one. Since the FI is an absolute measure and does not account for sample size, comparisons across different studies or establishing a set of reference values to categorise fragility levels proved difficult [16]. To overcome this, the fragility quotient (FQ) was proposed by Ahmed et al. in 2016 as a relative measure of trial robustness [17]. The FQ is simply the FI divided by the trial sample size, and thus expresses the proportion of trial participants whose outcome events would need to change to non-events to turn a trial result from ‘statistically significant’ to ‘statistically not significant’. Accordingly, the reverse fragility quotient (RFQ) is calculated by dividing the RFI by each trial’s total sample size.

Vaccines against SARS-CoV-2 have become one of the most important public health interventions around the world due to the COVID-19 pandemic. It is thus of utmost importance to establish how robust results of the underlying vaccine trials are. The use of FIs and FQs has become increasingly common in medical research, particularly in meta-analyses and systematic reviews [18-22], and as such constitute a promising method to improve our understanding about the robustness of COVID-19 vaccine trials. We therefore conducted a systematic review and meta-analysis of the global evidence with the aim to assess the robustness of COVID-19 vaccine efficacy (VE) trial results using the FI and FQ methodology. This study aims to provide valuable insights into the reliability of the scientific evidence about the efficacy of COVID-19 vaccines, which is crucial information for public health decision-making and communication with regards to COVID-19 vaccines.

## Methods

### Search strategy and selection criteria

This is a systematic review and meta-analysis of COVID-19 VE trial results. We registered the protocol of this study in the International Prospective Register of Systematic Reviews (PROSPERO) before commencing data collection (protocol number CRD42021258455) and followed the Cochrane Handbook for Systematic Reviews of Interventions [23] as well as the Preferred Reporting Items for Systematic Reviews and Meta-Analyses (PRISMA) [24,25] recommendations where applicable.

We included studies presenting results from RCTs in humans on the VE against SARS-CoV-2 that reported at least one dichotomous outcome based on frequentist statistics and were published in the scientific literature in English language before 22 January 2023. We excluded non-randomised studies, studies on animals, trials where VE was not a primary outcome, and trials that did not have either asymptomatic infection, morbidity (symptomatic infection), or mortality as outcome events. We also excluded publications that were not original research articles presenting new data, such as letters to the editor, viewpoints, and narrative reviews.

We searched the databases Medline (via PubMed), Cochrane Library, and Embase; Supplement File 2 presents detailed search statements for each database. References of relevant systematic reviews and of all included articles were also searched. All search results were uploaded into the Covidence software [26], where they were screened by two researchers (TNAH and HLQ) independently for relevance based on their titles and abstracts. Full texts of retained search results were retrieved, and the same pair of reviewers then independently reviewed full texts using a pre-established eligibility criteria checklist, which is described in Supplement File 3. At the titles/abstracts and full text screening stages, discrepancies were first discussed between the two researchers and, if necessary, resolved by a third researcher (FV). The reasons for exclusion were also documented, and a PRISMA flowchart of the selection process was constructed. One of the researchers (TNAH) extracted data from all included studies using a pre-established data extraction form, figuring in Supplement File 4. In this form, which we piloted before data extraction, data from trials with more than one primary outcome were extracted separately for each outcome. Another researcher (HLQ) reviewed a random sample of 20% (11/54) of included studies as a validity check, and discrepancies were discussed between the two researchers and resolved by a third reviewer (FV) if needed. Supplement File 5 lists some information on excluded studies.

### Data extraction

The following data were extracted from all included studies and entered into a dedicated study database: general information (authors, journal, month and year of publication); methods (study design, type of blinding, trial phase, allocation unit, randomisation ratio); participants (countries of patient enrolment, number of participants randomised, number of participants analysed); intervention and type of control (vaccine brand name, type of vaccine, type of control); type of outcome event (asymptomatic infection, symptomatic infection i.e. symptomatic disease of any severity, death); reported p value; reported VE including 95% confidence intervals; type of analysis (intention to treat, per protocol); and data required to calculate the FI and FQ (number of participants with outcome events in the intervention group, number of participants with outcome events in the control group, total number of participants in the intervention group, total number of participants in the control group). Separate records were created for each outcome for trials with more than one primary outcome.

### Risk of bias assessment

The risk of bias of included studies was assessed independently by two researchers (TNAH and HLQ) using the Cochrane Risk of Bias for Randomised Controlled Trials Tool version 2 (RoB2) [27]. RoB2 considers seven domains (random sequence generation, allocation concealment, blinding of participants and personnel, blinding of outcome assessment, incomplete outcome data, selective reporting, and other bias) and produces a summary assessment of the risk of bias categorised as low (low risk of bias in all domains), high (high risk of bias in at least one domain), or unclear (unclear risk of bias in at least one domain) [27].

### Data analysis

We calculated the FI and FQ for each primary outcome of all included studies according to the method described by Walsh et al. [13]. In short, the p value for each outcome was re-calculated using a two-sided Fisher exact test via the web interface of ClinCalc [28] by iteratively adding events to the group with the smaller number of events, while subtracting non-events from the same group to keep the total number of participants constant. Events were added one at a time until the calculated p value became equal to or larger than 0.05. The smallest number of additional outcome events required to obtain a p value of at least 0.05 constituted the FI for that trial outcome.

In the case of non-significant results, the RFI was calculated instead by subtracting events from the group with the smaller number of events while simultaneously adding non-events to the same group to maintain the total number of participants constant until the Fisher’s exact test p value became less than 0.05 [15].

We calculated the FQ by dividing the FI by the total number of participants that were included in the analysis (sample size) as described by Ahmed et al. [17]. On the other hand, the RFQ was calculated for all non-significant comparison arms. FQ and RFQ were presented as percentages for ease of interpretation [29].

All calculations were done by two reviewers (TNAH and HLQ) independently to minimise data entry and calculation errors.

Using the software package STATA v. 16.0 [30], we calculated the median and interquartile ranges (IQR) of FIs and FQs for risk of bias as per RoB2 classification; blinding type (single-blinded; double-or triple-blinded; open-label or not reported); magnitude of effect measure (VE < 70%; 70–90%; > 90%); trial phase; sample size (< 10,000; 10,000–25,000; > 25,000); and percentage loss to follow-up (LTFU) (< 5%; 5 – < 10%; 10 – < 15%; 15 – < 30%; ≥ 30%). We assessed the associations of trial characteristics with FI and FQ using Wilcoxon rank-sum tests and Kruskal–Wallis H tests. We produced scatter plots including least squares regression lines and used Spearman correlation coefficients to quantify the strength of correlation of the FI and FQ with sample size, percentage LTFU, and VE.

## Results

**Figure 1:**
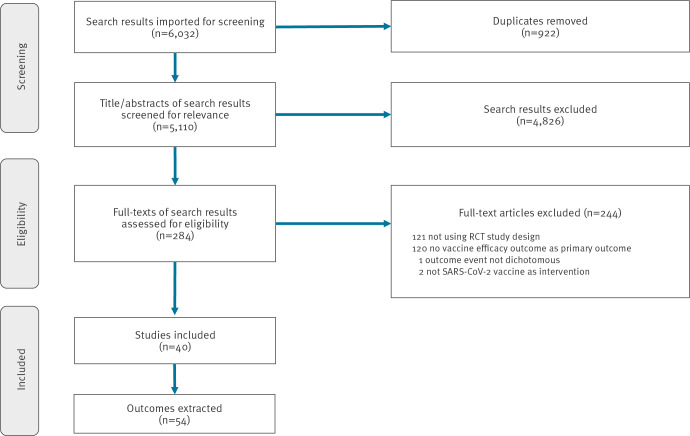
Selection of randomised controlled trial studies on COVID-19 vaccine efficacy, up to 22 January 2023 (n = 40 studies selected)

RCT: randomised controlled trial; SARS-CoV-2: severe acute respiratory syndrome coronavirus.

A total of 6,032 search results were retrieved from the three databases. After removing 922 duplicates, 5,110 titles/abstracts were screened for relevance, resulting in the exclusion of another 4,826 results. A further 244 records were excluded during the full text review for eligibility, mostly because they were not RCTs or did not have VE as primary outcome. Ultimately, a total of 54 primary outcomes from 40 included studies were identified and their data extracted for analysis (Figure 1).

Eleven (28%) of the 40 included studies reported on more than one primary outcome, resulting in 54 outcomes for our analysis with a total of 909,404 participants (median:  13,993; IQR: 8,534–25,519). The randomisation ratio was 1:1 in 45 (83%) outcomes. Twelve (22%) outcomes assessed the efficacy of Vaxzevria (ChAdOx1/nCoV-19, AstraZeneca, Cambridge, United Kingdom), 11 (20%) Comirnaty (BNT162b2, BioNTech/Pfizer, Mainz, Germany/New York, United States), five (9%) the Janssen COVID-19 vaccine (Ad26.COV2-S, Janssen–Cilag International NV, Beerse, Belgium) and 26 (48%) other vaccines. Most included outcomes were from studies in trial phase 3 (38/47, 81%). Nearly half of the outcomes (24/54, 44%) were from double-blinded or triple-blinded studies. Twenty-one (39%) and 17 (31%) outcomes were based on sample sizes under 10,000 or over 25,000 participants, respectively. Twenty (37%) and 18 (33%) outcomes reported a VE of < 70% and > 90%, respectively. Fifteen (29%) outcomes had the percentage LTFU between 5% and 10% (Table 1, Table 2 and Table 3).

**Table 1:** Characteristics of primary outcomes (n = 54) of COVID-19 vaccine efficacy studies included in this analysis up to 22 January 2023 (n = 40 studies)

Outcome ID^a^	Authors	Journal of publication	Time of publication	Study design	Blinding type	Trial phase	Randomisation ratio (allocation unit)	Countries of enrolment	Vaccine name	Vaccine type/ control type	Type of outcome	Reported p value	Reported vaccine efficacy (95%CI)^b^	Type of analyses
1	Voysey et al. [49]	Lancet	Feb 2021	Superiority with parallel groups	Patient-blinded and clinician-blinded	1/2, 2/3, 3	1:1 (individual)	UK, Brazil, South Africa	Vaxzevria (ChAdOx1/nCoV-19, AstraZeneca, Cambridge, UK)	Viral vector/ placebo	Symptomatic infection	Not reported	66.7 (57.4 to 74.0)	Per protocol
2.1	Kaabi et al.^c,d^ [50]	JAMA	May 2021	Superiority with parallel groups	Patient-blinded and clinician-blinded	3	1:1 (individual)	UAE, Bahrain	SARS-CoV-2 WIV04 vaccine (Wuhan COVID-19 Vaccine, China National Biotec Group Company Limited, Beijing, China)	Inactivated/ placebo	Symptomatic infection	< 0.001	72.8 (58.1 to 82.4)	Per protocol
2.2	Kaabi et al.^c,d^ [50]	JAMA	May 2021	Superiority with parallel groups	Patient-blinded and clinician-blinded	3	1:1 (individual)	UAE, Bahrain	HB02 vaccine (Wuhan COVID-19 Vaccine, China National Biotec Group Company Limited, Beijing, China)	Inactivated/ placebo	Symptomatic infection	< 0.001	78.1 (64.8 to 86.3)	Per protocol
3	Baden et al. [51]	NEJM	Feb 2021	Superiority with parallel groups	Clinician-blinded	3	1:1 (individual)	US	Spikevax (mRNA-1273, Moderna, Cambridge, US)	mRNA/ placebo	Symptomatic infection	< 0.001	94.1 (89.3 to 96.8)	Per protocol
4	Emary et al. [52]	Lancet	Mar 2021	Superiority with parallel groups	Patient-blinded	2/3	1:1 (individual)	England, Wales, Scotland	Vaxzevria (ChAdOx1/nCoV-19, AstraZeneca, Cambridge, UK)	Viral vector/ placebo	Symptomatic infection	Not reported	72.3 (63.1 to 79.3)	Not reported
5.1	Frenck et al.^d^ [53]	NEJM	May 2021	Superiority with parallel groups	Not reported	3	1:1 (individual)	US	Comirnaty (BNT162b2 mRNA, BioNTech/Pfizer, Mainz, Germany/New York, US)	mRNA/ placebo	Symptomatic infection	Not reported	100 (75.3 to 100)	Not reported
5.2	Frenck et al.^d^ [53]	NEJM	May 2021	Superiority with parallel groups	Not reported	3	1:1 (individual)	US	Comirnaty (BNT162b2 mRNA, BioNTech/Pfizer, Mainz, Germany/New York, US)	mRNA/ placebo	Symptomatic infection	Not reported	100 (78.1 to 100)	Not reported
6	Heath et al. [54]	NEJM	Jun 2021	Superiority with parallel groups	Not reported	3	1:1 (individual)	UK	Nuvaxovid (NVX-CoV2373, Novavax/CEPI, Gaithersburg, US/Oslo, Norway)	Recombinant/ placebo	Symptomatic infection	Not reported	89.7 (80.2 to 94.6)	Per protocol
7	Logunov et al. [55]	Lancet	Feb 2021	Superiority with parallel groups	Patient-blinded and clinician-blinded	3	3:1 (individual)	Russia	Sputnik V (Gam-COVID-Vac, Russia)	Viral vector/ placebo	Asymptomatic or symptomatic infection	< 0.0001	91.6 (85.6 to 95.2)	Per protocol
8	Madhi et al. [56]	NEJM	May 2021	Superiority with parallel groups	Patient-blinded and clinician-blinded	1b-2	1:1 (individual)	South Africa	Vaxzevria (ChAdOx1/nCoV-19, AstraZeneca, Cambridge, UK)	Viral vector/ placebo	Symptomatic infection	Not reported	21.9 (−49.9 to 59.8)	Per protocol
9.1	Polack et al.^d^ [57]	NEJM	Dec 2020	Superiority with parallel groups	Not reported	2/3	1:1 (individual)	US, Argentina, Brazil, South Africa, Germany, Turkey	Comirnaty (BNT162b2 mRNA, BioNTech/Pfizer, Mainz, Germany/New York, US)	mRNA/ placebo	Symptomatic infection	Not reported	95 (90.3 to 97.6)	Not reported
9.2	Polack et al.^d^ [57]	NEJM	Dec 2020	Superiority with parallel groups	Not reported	2/3	1:1 (individual)	US, Argentina, Brazil, South Africa, Germany, Turkey	Comirnaty (BNT162b2 mRNA, BioNTech/Pfizer, Mainz, Germany/New York, US)	mRNA/ placebo	Symptomatic infection	Not reported	94.6 (89.9 to 97.3)	Not reported
10.1	Sadoff et al.^d^ [58]	NEJM	Apr 2021	Superiority with parallel groups	Patient-blinded and clinician-blinded	3	1:1 (individual)	Argentina, Brazil, Chile, Colombia, Mexico, Peru, South Africa, US	Janssen vaccine (Ad26.COV2-S, Janssen–Cilag International NV, Beerse, Belgium)	Viral vector/ placebo	Symptomatic infection	Not reported	66.9 (59 to 73.4)	Per protocol
10.2	Sadoff et al.^d^ [58]	NEJM	Apr 2021	Superiority with parallel groups	Patient-blinded and clinician-blinded	3	1:1 (individual)	Argentina, Brazil, Chile, Colombia, Mexico, Peru, South Africa, US	Janssen vaccine (Ad26.COV2-S, Janssen–Cilag International NV, Beerse, Belgium)	Viral vector/ placebo	Symptomatic infection	Not reported	66.1 (55 to 74.8)	Per protocol
11	Shinde et al. [59]	NEJM	May 2021	Superiority with parallel groups	Patient-blinded and clinician-blinded	2a-b	1:1 (individual)	South Africa	Nuvaxovid (NVX-CoV2373, Novavax/CEPI, Gaithersburg, US/Oslo, Norway)	Recombinant/ placebo	Symptomatic infection	Not reported	49.4 (6.1 to 72.8)	Per protocol
12	Tanriover et al. [60]	Lancet	July 2021	Superiority with parallel groups	Patient-blinded and clinician-blinded	3	2:1 (individual)	Turkey	CoronaVac vaccine (Sinovac Life Sciences, Beijing, China)	Inactivated/ placebo	Symptomatic infection	< 0.0001	83.5 (65.4 to 92.1)	Per protocol
13	Voysey et al. [61]	Lancet	Dec 2020	Superiority with parallel groups	Patient-blinded and clinician-blinded	1/2, 2/3, 3	1:1 (individual)	UK, Brazil, South Africa	Vaxzevria (ChAdOx1/nCoV-19, AstraZeneca, Cambridge, UK)	Viral vector/ placebo	Symptomatic infection	Not reported	70.4 (54.8 to 80.6)	Per protocol
14	Thomas et al.^d^ [62]	NEJM	Sep 2021	Superiority with parallel groups	Open-label	2/3	1:1 (individual)	US, Argentina, Brazil, South Africa, Germany, Turkey	Comirnaty (BNT162b2 mRNA, BioNTech/Pfizer, Mainz, Germany/New York, US)	mRNA/ placebo	Symptomatic infection	Not reported	91.3 (89 to 93.2)	Not reported
15.1	Sadoff et al.^d^ [63]	NEJM	Sep 2021	Superiority with crossover groups	Patient-blinded and clinician-blinded		1:1 (individual)	Argentina, Brazil, Chile, Colombia, Mexico, Peru, South Africa, US	Janssen vaccine (Ad26.COV2-S, Janssen–Cilag International NV, Beerse, Belgium)	Viral vector/ placebo	Symptomatic infection	Not reported	56.3 (51.3 to 60.8)	Per protocol
15.2	Sadoff et al.^d^ [63]	NEJM	Sep 2021	Superiority with crossover groups	Patient-blinded and clinician-blinded	3	1:1 (individual)	Argentina, Brazil, Chile, Colombia, Mexico, Peru, South Africa, US	Janssen vaccine (Ad26.COV2-S, Janssen–Cilag International NV, Beerse, Belgium)	Viral vector/ placebo	Symptomatic infection	Not reported	52.9 (47.1 to 58.1)	Per protocol
16	Kremsner et al. [64]	Lancet Infectious Diseases	Nov 2021	Superiority with parallel groups	Clinician-blinded and statistician-blinded	2/3	1:1 (individual)	Belgium, Germany, the Netherlands, Spain, Argentina, Colombia, Dominican Republic, Mexico, Panama, Peru	CVnCoV (CureVac N.V./CEPI, Tübingen, Germany /Oslo, Norway)	mRNA/ placebo	Symptomatic infection	0.016	48.2 (95.826% CI: 31 to 61.4)	Per protocol
17	Khobragade et al. [65]	Lancet	Apr 2022	Superiority with parallel groups	Patient-blinded and clinician-blinded	3	1:1 (individual)	India	ZyCoV-D (Cadila Healthcare, Ahmedabad, India)	DNA-based/ placebo	Symptomatic infection	Not reported	66.6 (47.6 to 80.7)	Per protocol
18	Falsey et al. [66]	NEJM	Sep 2021	Superiority with parallel groups	Not reported	3	2:1 (individual)	US, Chile, Peru	Vaxzevria (ChAdOx1/nCoV-19, AstraZeneca, Cambridge, UK)	Viral vector/ placebo	Symptomatic infection	< 0.001	74 (65.3 to 80.5)	Not reported
19	Fadlyana et al. [67]	Vaccine	Sep 2021	Superiority with parallel groups	Not reported	3	1:1 (individual)	Indonesia	CoronaVac vaccine (Sinovac Life Sciences, Beijing, China)	Inactivated/ placebo	Symptomatic infection	Not reported	65.3 (CI not reported)	Not reported
20	Ella et al. [68]	Lancet	Nov 2021	Superiority with parallel groups	Patient-blinded and clinician-blinded	3	1:1 (individual)	India	Covaxin (BBV152, Bharat Biotech, Turakapally, India)	Inactivated/ placebo	Symptomatic infection	Not reported	77.8 (65.2 to 86.4)	Per protocol
21	El Sahly et al. [69]	NEJM	Sep 2021	Superiority with parallel groups	Clinician-blinded	3	1:1 (individual)	US	Spikevax (mRNA-1273, Moderna, Cambridge, US)	mRNA/ placebo	Symptomatic infection	Not reported	93.2 (91 to 94.8)	Per protocol
22.1	Clemens et al.^d^ [70]	Nature	Oct 2021	Superiority with parallel groups	Patient-blinded and clinician-blinded	3	1:1 (individual)	Brazil	Vaxzevria (ChAdOx1/nCoV-19, AstraZeneca, Cambridge, UK)	Viral vector/ placebo	Symptomatic infection	Not reported	88.2 (5.4 to 98.5)	Not reported
22.2	Clemens et al.^d^ [70]	Nature	Oct 2021	Superiority with parallel groups	Patient-blinded and clinician-blinded	3	1:1 (individual)	Brazil	Vaxzevria (ChAdOx1/nCoV-19, AstraZeneca, Cambridge, UK)	Viral vector/ placebo	Symptomatic infection	Not reported	72.6 (46.4 to 86)	Not reported
22.3	Clemens et al.^d^ [70]	Nature	Oct 2021	Superiority with parallel groups	Patient-blinded and clinician-blinded	3	1:1 (individual)	Brazil	Vaxzevria (ChAdOx1/nCoV-19, AstraZeneca, Cambridge, UK)	Viral vector/ placebo	Symptomatic infection	Not reported	68.7 (54.9 to 78.3)	Not reported
22.4	Clemens et al.^d^ [70]	Nature	Oct 2021	Superiority with parallel groups	Patient-blinded and clinician-blinded	3	1:1 (individual)	Brazil	Vaxzevria (ChAdOx1/nCoV-19, AstraZeneca, Cambridge, UK)	Viral vector/ placebo	Symptomatic infection	Not reported	63.6 (−2.1 to 87)	Not reported
23.1	Walter et al.^d^ [71]	NEJM	Nov 2021	Superiority with parallel groups	Patient-blinded	2/3	2:1 (individual)	US, Spain, Finland, Poland	Comirnaty (BNT162b2 mRNA, BioNTech/Pfizer, Mainz, Germany/New York, US)	mRNA/ placebo	Symptomatic infection	Not reported	90.7 (67.7 to 98.3)	Not reported
23.2	Walter et al.^d^ [71]	NEJM	Nov 2021	Superiority with parallel groups	Patient-blinded	2/3	2:1 (individual)	US, Spain, Finland, Poland	Comirnaty (BNT162b2 mRNA, BioNTech/Pfizer, Mainz, Germany/New York, US)	mRNA/ placebo	Symptomatic infection	Not reported	90.7 (67.4 to 98.3)	Not reported
24.1	Thomas et al.^d^ [72]	Vaccine	Dec 2021	Superiority with parallel groups	Not reported	3	1:1 (individual)	US, Argentina, Brazil, South Africa, Germany, Turkey	Comirnaty (BNT162b2 mRNA, BioNTech/Pfizer, Mainz, Germany/New York, US)	mRNA/ placebo	Symptomatic infection	Not reported	94.4 (85.1 to 98.5)	Not reported
24.2	Thomas et al.^d^ [72]	Vaccine	Dec 2021	Superiority with parallel groups	Not reported	3	1:1 (individual)	US, Argentina, Brazil, South Africa, Germany, Turkey	Comirnaty (BNT162b2 mRNA, BioNTech/Pfizer, Mainz, Germany/New York, US)	mRNA/ placebo	Symptomatic infection	Not reported	94.4 (85.2 to 98.5)	Not reported
25	Pajon et al. [73]	Nature Medicine	Apr 2022	Superiority with parallel groups	Clinician-blinded	3	1:1 (individual)	US	Spikevax (mRNA-1273, Moderna, Cambridge, US)	mRNA/ placebo	Symptomatic infection	Not reported	93.2 (91 to 94.8)	Per protocol
26.1	Moreira et al.^d^ [74]	NEJM	Mar 2022	Superiority with parallel groups	Patient-blinded	3	1:1 (individual)	US, South Africa, Brazil	Comirnaty (BNT162b2 mRNA, BioNTech/Pfizer, Mainz, Germany/New York, US)	mRNA/ placebo	Symptomatic infection	Not reported	95.3 (89.5 to 98.3)	Not reported
26.2	Moreira et al.^d^ [74]	NEJM	Mar 2022	Superiority with parallel groups	Patient-blinded	3	1:1 (individual)	US, South Africa, Brazil	Comirnaty (BNT162b2 mRNA, BioNTech/Pfizer, Mainz, Germany/New York, US)	mRNA/ placebo	Symptomatic infection	Not reported	94.6 (88.5 to 97.9)	Not reported
27	Halperin et al. [75]	Lancet	Dec 2021	Superiority with parallel groups	Patient-blinded and clinician-blinded	3	1:1 (individual)	Argentina, Chile, Mexico, Pakistan, Russia	Convidecia (Ad5-nCoV vaccine, CanSino, Tianjin, China)	Viral vector/ placebo	Symptomatic infection	0.0026	57.5 (39.7 to 70)	Intention to treat
28	Dunkle et al. [76]	NEJM	Dec 2021	Superiority with parallel groups	Clinician-blinded	3	2:1 (individual)	US and Mexico	Nuvaxovid (NVX-CoV2373, Novavax/CEPI, Gaithersburg, US/Oslo, Norway)	Recombinant/ placebo	Symptomatic infection	< 0.001	90.4 (82.9 to 94.6)	Per protocol
29	Bravo et al. [77]	Lancet	Jan 2022	Superiority with parallel groups	Patient-blinded and clinician-blinded and statistician-blinded	3	1:1 (individual)	Belgium, Brazil, Colombia, Philippines, South Africa	SCB-2019 (Clover Biopharmaceuticals, Chengdu Shi, China)	Recombinant/ placebo	Asymptomatic or symptomatic infection	Not reported	67.2 (95.72% CI: 54.3 to 76.8)	Per protocol
30	Wang et al. [78]	Emerging Microbes and Infections	Jun 2022	Superiority with parallel groups	Unclear	3	1:1 (individual)	Pakistan, Malaysia	V-01 (Livzon Pharmaceutical Group Inc. Zhuhai city, China)	Protein subunit/ placebo	Symptomatic infection	Not reported	47.8 (22.6 to 64.7)	Modified intention to treat
31	Tanriover et al. [79]	Vaccines	Nov 2022	Non-superiority with parallel groups	Unclear	3	1:1 (individual)	Türkiye	TURKOVAC (Health Institutes of Turkey, Turkey)	Inactivated/ coronavac	Symptomatic infection	Not reported	41.03 (12.95 to 60.06)	Modified intention to treat
32	Tabarsi et al. [80]	Clinical Microbiology and Infection	Sep 2022	Superiority with parallel groups	Patient-blinded and clinician-blinded	3	3:1 (individual)	Iran	SpikoGen (Vaxine/CinnaGen Co., Adelaide, Australia/Tehran, Iran)	Protein subunit/ placebo	Symptomatic infection	Not reported	43.99 (30.3 to 55)	Per protocol
33	Sobieszczyk et al. [81]	Clinical Investigation	Sep 2022	Superiority with parallel groups	Patient-blinded and clinician-blinded	3	2:1 (individual)	US, Chile, Peru	Vaxzevria (ChAdOx1/nCoV-19, AstraZeneca, Cambridge, UK)	Viral vector/ placebo	Symptomatic infection	< 0.001	67 (58.9 to 73.5)	Not mentioned
34.1	Smolenov et al.^d^ [82]	Lancet Infectious Diseases	Nov 2021	Superiority with parallel groups	Clinician-blinded	2, 3	1:1 (individual)	Belgium, Brazil, Colombia, The Philippines, South Africa	SCB-2019 (Clover Biopharmaceuticals, Chengdu Shi, China)	Recombinant/ placebo	Symptomatic infection	Not reported	83.2 (78 to 87.3)	Not mentioned
34.2	Smolenov et al.^d^ [82]	Lancet Infectious Diseases	Nov 2021	Superiority with parallel groups	Clinician-blinded	2, 3	1:1 (individual)	Belgium, Brazil, Colombia, The Philippines, South Africa	SCB-2019 (Clover Biopharmaceuticals, Chengdu Shi, China)	Recombinant/ placebo	Symptomatic infection	Not reported	92.5 (82.9 to 97.3)	Not mentioned
34.3	Smolenov et al.^d^ [82]	Lancet Infectious Diseases	Nov 2021	Superiority with parallel groups	Clinician-blinded	2, 3	1:1 (individual)	Belgium, Brazil, Colombia, The Philippines, South Africa	SCB-2019 (Clover Biopharmaceuticals, Chengdu Shi, China)	Recombinant/ placebo	Symptomatic infection	Not reported	100 (59.3 to 100)	Not mentioned
35.1	Marchevsky et al.^d^ [83]	Lancet	Jul 2022	Superiority with parallel groups	Patient-blinded	2/3, 3	1:1 (individual)	UK, Brazil	Vaxzevria (ChAdOx1/nCoV-19, AstraZeneca, Cambridge, UK)	Viral vector/ placebo	Symptomatic infection	Not reported	59.9 (49.8 to 67.9)	Per protocol
35.2	Marchevsky et al.^d^ [83]	Lancet	Jul 2022	Superiority with parallel groups	Patient-blinded	2/3, 3	1:1 (individual)	UK, Brazil	Vaxzevria (ChAdOx1/nCoV-19, AstraZeneca, Cambridge, UK)	Viral vector/ placebo	Symptomatic infection	Not reported	66.1 (55.9 to 73.9)	Per protocol
36	Khairullin et al. [84]	Lancet	Jun 2022	Superiority with parallel groups	Unclear	3	4:1 (individual)	Kazakhstan	QazVac (QazCovid-in, Kazakhstan)	Inactivated/ placebo	Symptomatic infection	Not reported	82 (71.1 to 88.5)	Intention to treat
37	Heath et al. [85]	Clinical Infectious Diseases	Oct 2022	Superiority with parallel groups	Patient-blinded	3	1:1 (individual)	UK	Nuvaxovid (NVX-CoV2373, Novavax/CEPI, Gaithersburg, US/Oslo, Norway)	Recombinant/ placebo	Symptomatic infection	Not reported	82.7 (73.3 to 88.8)	Per protocol
38	Hardt et al. [86]	Lancet Infectious Diseases	Nov 2021	Superiority with crossover groups	Unclear	3	1:1 (individual)	Belgium, Brazil, Colombia, France, Germany, the Philippines, South Africa, Spain, UK, US	Janssen vaccine (Ad26.COV2-S, Janssen–Cilag International NV, Beerse, Belgium)	Viral vector/ placebo	Symptomatic infection	Not reported	75.6 (55.5 to 87.5)	Per protocol
39	Hager et al. [87]	NEJM	May 2022	Superiority with parallel groups	Unclear	3	1:1 (individual)	Argentina, Brazil, Canada, Mexico, UK, US	Covifenz (CoVLP + AS03, Mitzubishi, Tokyo, Japan)	Virus-like particles/ placebo	Symptomatic infection	Not reported	71 (58.7 to 80)	Per protocol
40	Dai et al. [88]	NEJM	May 2022	Superiority with parallel groups	Patient-blinded and clinician-blinded	3	1:1 (individual)	Uzbekistan, Indonesia, Pakistan, Ecuador	Zifivax or ZF-UZ-VAC-2001 (ZF2001, Anhui Zhifei Longcom, Anhui, China)	Protein subunit/ placebo	Symptomatic infection	Not reported	81.4 (73.3 to 87.3)	Intention to treat

CEPI: Coalition for Epidemic Preparedness Innovations; CI: confidence interval; ID: identifier; JAMA: Journal of the American Medical Association; NEJM: New England Journal of Medicine; UAE: United Arab Emirates; UK: United Kingdom; US: United States of America.

^a^ Each of the 40 studies is given a number from 1 to 40. The outcome ID of a single-outcome study is identical to the study number (e.g. the outcome of ‘study 1’ is called ‘1’). If a study has more than one primary outcome, each of its outcomes are given two digits; the first digit is the study number and the second, the primary outcome number (e.g. for ‘study 2’ the first outcome’ is designated ‘2.1’, the second outcome ‘2.2’).

^b^ The confidence intervals (CI) presented in brackets in this column are 95% confidence intervals unless specified otherwise.

^c^ This study assessed two interventions.

^d^ These studies had more than one primary outcome.

**Table 2:** Fragility index and fragility quotient according to the characteristics of primary outcomes (n = 54) of studies included in this analysis up to 22 January 2023 (n = 40 studies)

Characteristics	Number of outcomes (%)	Median FI (IQR)	p value^a^	Median FQ % (IQR)	p value^a^
**Total**	**54 (100)**	**62 (22–123)**	**NA**	**0.50 (0.24–0.92)**	**NA**
Risk of bias					
Low risk	3 (6)	44 (24–49)	0.276	0.19 (0.17–0.21)	0.040
High risk	32 (59)	88 (13–142)		0.55 (0.34–1.28)	
Unclear	19 (35)	52 (22–88)		0.32 (0.13–0.67)	
Blinding type					
Single-blinded	15 (28)	87 (46–139)	0.332	0.87 (0.49–1.53)	0.023
Double- or triple-blinded	24 (44)	51 (26–124)		0.36 (0.16–0.62)	
Open-label or not reported	15 (28)	45 (8–119)		0.36 (0.31–1.24)	
Vaccine efficacy					
< 70%	20 (37)	62 (11–120)	0.780	0.53 (0.14–0.75)	0.335
70–90%	16 (30)	58 (37–102)		0.36 (0.20–0.94)	
> 90%	18 (33)	85 (12–125)		0.58 (0.33–1.24)	
Trial phase^b^					
1/2 or 2	2 (4)	5 (1–8)	0.120	0.30 (0.04–0.55)	0.576
2/3	7 (15)	111 (12–125)		0.55 (0.31–1.30)	
3	38 (81)	62 (24–125)		0.48 (0.21–0.87)	
Sample size					
< 10,000	21 (39)	12 (7–86)	< 0.001	0.55 (0.13–1.24)	0.478
10,000–25,000	16 (30)	58 (32–84)		0.36 (0.25–0.62)	
> 25,000	17 (31)	144 (92–380)		0.49 (0.31–1.28)	
Percentage of loss to follow-up^c^					
< 5%	11 (21)	45 (12–166)	0.561	0.55 (0.29–1.38)	0.299
5 – < 10%	15 (29)	61 (44–125)		0.49 (0.17–0.92)	
10 – < 15%	8 (15)	137 (17–371)		0.54 (0.30–1.14)	
15 – < 30%	9 (17)	88 (62–119)		0.55 (0.33–0.72)	
≥ 30%	9 (17)	37 (24–55)		0.21 (0.15–0.32)	

FI: fragility index; FQ: fragility quotient; IQR: interquartile range; LFTU: loss to follow-up; NA: not applicable.

^a^ p values from Kruskal–Wallis H test.

^b^ We excluded seven outcomes from four studies that could not be categorised into any of the trial phases.

^c^ We excluded two outcomes from two studies because they did not report the denominator of percentage LTFU, i.e. the number of participants randomised.

**Table 3:** Parameters for calculating the fragility index, reverse fragility index and fragility quotient and presentation of the values obtained for each primary outcome (n = 54) of studies included in this analysis up to 22 January 2023 (n = 40 studies)

Outcome ID^a^	Authors	Participants randomised	Participants analysed	Loss to follow-up	Participants with outcome events in intervention group	Participants with outcome events in control group	Total participants in intervention group	Total participants in control group	Recalculated p value	FI/RFI	FQ/RFQ	FQ (%)
1	Voysey et al. [49]	24,422	17,178	7,244	84	248	8,597	8,581	< 0.001	123	0.0072	0.72
2.1	Kaabi et al.^b,c^ [50]	26,941	25,536	1,405	26	95	12,769	12,767	< 0.001	44	0.0017	0.17
2.2	Kaabi et al. ^b,c^ [50]	26,941	25,519	1,422	21	95	12,752	12,767	< 0.001	49	0.0019	0.19
3	Baden et al. [51]	30,420	28,207	2,213	11	185	14,134	14,073	< 0.001	139	0.0049	0.49
4	Emary et al. [52]	Not reported	8,534	Not reported	59	210	4,244	4,290	< 0.001	111	0.013	1.3
5.1	Frenck et al.^c^ [53]	2,264	1,983	281	0	16	1,005	978	< 0.001	7	0.0035	0.35
5.2	Frenck et al.^c^ [53]	2,264	2,229	35	0	18	1,119	1,110	< 0.001	8	0.0036	0.36
6	Heath et al. [54]	15,187	14,039	1,148	10	96	7,020	7,019	< 0.001	61	0.0043	0.43
7	Logunov et al. [55]	21,977	19,866	2,111	16	62	14,964	4,902	< 0.001	125	0.0063	0.63
8	Madhi et al.^d^ [56]	2,026	1,467	559	19	23	750	717	0.532	8	0.0055	0.55
9.1	Polack et al.^c^ [57]	43,548	36,523	7,025	8	162	18,198	18,325	< 0.001	119	0.0033	0.33
9.2	Polack et al.^c^ [57]	43,548	40,137	3,411	9	169	19,965	20,172	< 0.001	125	0.0031	0.31
10.1	Sadoff et al.^c^ [58]	44,325	39,058	5,267	116	348	19,514	19,544	< 0.001	182	0.0047	0.47
10.2	Sadoff et al.^c^ [58]	44,325	38,484	5,841	66	193	19,306	19,178	< 0.001	92	0.0024	0.24
11	Shinde et al. [59]	4,406	2,684	1,722	15	29	1,357	1,327	0.033	1	0.0004	0.04
12	Tanriover et al. [60]	10,218	10,029	189	9	32	6,559	3,470	< 0.001	29	0.0029	0.29
13	Voysey et al. [61]	Not reported	11,636	Not reported	30	101	5,807	5,829	< 0.001	45	0.0039	0.39
14	Thomas et al.^c^ [62]	44,165	42,094	2,071	77	850	20,998	21,096	< 0.001	692	0.0164	1.64
15.1	Sadoff et al.^c^ [63]	43,788	38,798	4,990	484	1,067	19,400	19,398	< 0.001	497	0.0128	1.28
15.2	Sadoff et al.^c^ [63]	43,788	38,037	5,751	433	883	19,113	18,924	< 0.001	380	0.0100	1.00
16	Kremsner et al. [64]	39,680	25,062	14,618	83	145	12,851	12,211	< 0.001	37	0.0015	0.15
17	Khobragade et al. [65]	27,703	24,670	3,033	20	61	12,350	12,320	< 0.001	22	0.0009	0.09
18	Falsey et al. [66]	32,451	26,212	6,239	73	130	17,662	8,550	< 0.001	144	0.0055	0.55
19	Fadlyana et al. [67]	1,620	1,620	0	7	18	811	809	0.028	1	0.0006	0.06
20	Ella et al. [68]	25,798	16,973	8,825	24	106	8,471	8,502	< 0.001	55	0.0032	0.32
21	El Sahly et al. [69]	30,415	28,451	1,964	55	744	14,287	14,164	< 0.001	623	0.0219	2.19
22.1	Clemens et al.^c^ [70]	10,416	9,433	983	1	8	4,772	4,661	0.02	1	0.0001	0.01
22.2	Clemens et al.^c^ [70]	10,416	9,433	983	11	38	4,772	4,661	< 0.001	12	0.0013	0.13
22.3	Clemens et al.^c^ [70]	10,416	9,433	983	38	115	4,772	4,661	< 0.001	52	0.0055	0.55
22.4	Clemens et al.^c,d^ [70]	10,416	9,433	983	5	13	4,772	4,661	0.061	1	0.0001	0.01
23.1	Walter et al.^c^ [71]	2,285	1,968	317	3	16	1,305	663	< 0.001	12	0.0061	0.61
23.2	Walter et al.^c^ [71]	2,285	2,186	99	3	16	1,450	736	< 0.001	12	0.0055	0.55
24.1	Thomas et al.^c^ [72]	3,813	3,538	275	4	71	1,750	1,788	< 0.001	45	0.0127	1.27
24.2	Thomas et al.^c^ [72]	3,813	3,636	177	4	71	1,802	1,834	< 0.001	45	0.0124	1.24
25	Pajon et al. [73]	28,451	28,451	0	55	744	14,287	14,164	< 0.001	623	0.0219	2.19
26.1	Moreira et al.^c^ [74]	10,136	9,366	770	6	123	4,695	4,671	< 0.001	86	0.0092	0.92
26.2	Moreira et al.^c^ [74]	10,136	9,945	191	7	124	4,993	4,952	< 0.001	87	0.0087	0.87
27	Halperin et al. [75]	36,982	21,250	15,732	45	105	10,660	10,590	< 0.001	34	0.0016	0.16
28	Dunkle et al. [76]	29,949	25,452	4,497	14	63	17,312	8,140	< 0.001	83	0.0033	0.33
29	Bravo et al. [77]	30,174	11,741	18,433	52	155	5,935	5,806	< 0.001	71	0.006	0.6
30	Wang et al. [78]	10,241	9,869	372	38	72	4,935	4,934	0.001	13	0.0013	0.13
31	Tanriover et al. [79]	1,290	915	375	35	61	456	459	0.007	7	0.0077	0.77
32	Tabarsi et al. [80]	16,876	13,067	3,809	247	119	9,998	3,069	< 0.001	88	0.0067	0.67
33	Sobieszczyk et al. [81]	32,450	32,380	70	141	184	21,583	10,797	< 0.001	166	0.0051	0.51
34.1	Smolenov et al.^c^ [82]	30,174	14,670	15,504	65	353	7,339	7,331	< 0.001	239	0.0163	1.63
34.2	Smolenov et al.^c^ [82]	30,174	14,670	15,504	6	73	7,339	7,331	< 0.001	46	0.0031	0.31
34.3	Smolenov et al.^c^ [82]	30,174	14,670	15,504	0	10	7,339	7,331	0.002	3	0.0002	0.02
35.1	Marchevsky et al.^c^ [83]	8,528	8,393	135	109	261	4,243	4,150	< 0.001	116	0.0138	1.38
35.2	Marchevsky et al.^c^ [83]	8,528	6,776	1,752	75	218	3,376	3,400	< 0.001	104	0.0153	1.53
36	Khairullin et al. [84]	3,000	2,835	165	31	43	2,275	560	< 0.001	93	0.0328	3.28
37	Heath et al. [85]	15,185	13,946	1,239	24	134	6,961	6,985	< 0.001	80	0.0057	0.57
38	Hardt et al. [86]	31,300	11,639	19,661	14	53	6,024	5,615	< 0.001	24	0.0021	0.21
39	Hager et al. [87]	24,141	20,090	4,051	39	118	10,554	9,536	< 0.001	62	0.0031	0.31
40	Dai et al. [88]	28,904	25,193	3,711	158	580	12,625	12,568	< 0.001	361	0.0143	1.43

FI: fragility index; FQ: fragility quotient; ID: identifier; RFI: reverse fragility index.

^a^ Each of the 40 studies is given a number from 1 to 40. The outcome ID of a single-outcome study is identical to the study number (e.g. the outcome of ‘study 1’ is called ‘1’). If a study has more than one primary outcome, each of its outcomes are given two digits; the first digit is the study number and the second, the primary outcome number (e.g. for ‘study 2’ the first outcome’ is designated ‘2.1’, the second outcome ‘2.2’).

^b^ This study assessed two interventions.

^c^ These studies had more than one primary outcome.

^d^ RFI and RFQ were applied for these studies with p value exceeding 0.05.

Overall, the median FI was 62 (IQR: 22–123; range: 0–692) and the median FQ was 0.50% (IQR: 0.24–0.92; range: 0–3.28). The FI was not associated with trial characteristics at statistically significant levels other than trial sample size, for which there was a positive correlation. For a size < 10,000, a median FI of 12 (IQR: 7–86) was observed, while for a size range of 10,000–25,000, the median FI was 58 (IQR: 32–84) and for a size > 25,000, this was 144 (IQR: 92–380); p value < 0.001. The FQ was associated with blinding type at statistically significant levels: for single-blinded the median FQ was 0.87 (IQR: 0.49–1.53), double-or triple blinded, 0.36 (IQR: 0.16–0.62), open-label/not reported, 0.36 (QR: 0.31–1.24), p value 0.023 (Table 2). The number of participants LTFU exceeded the FI in 51 (94%) outcomes (Table 3).

**Figure 2:**
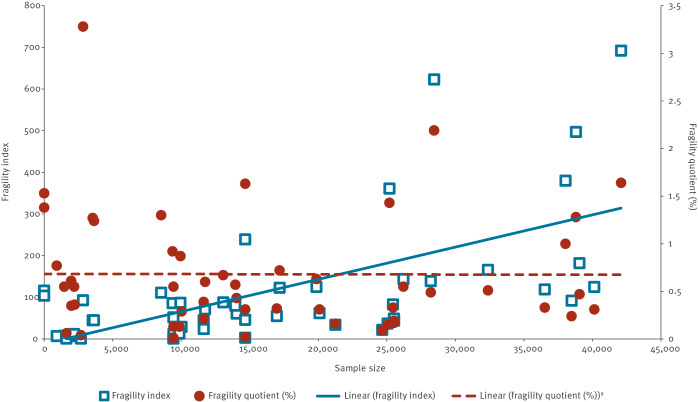
Fragility index and fragility quotient of outcomes against sample size, for studies included in this analysis up to 22 January 2023 (n = 40 studies selected)

^a^ Fitted least squares regression line.

**Figure 3:**
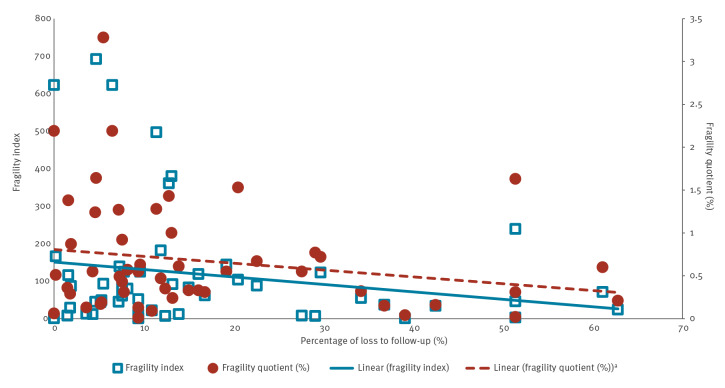
Fragility index and fragility quotient of outcomes against percentage lost to follow-up, for studies included in this analysis up to 22 January 2023 (n = 40 studies selected)

^a^ Fitted least squares regression line.

**Figure 4:**
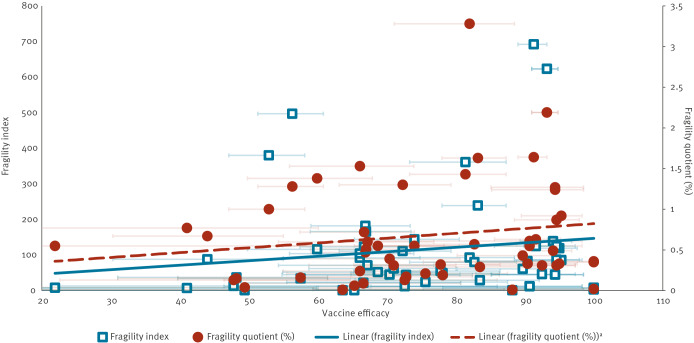
Fragility index and fragility quotient for outcomes, against vaccine efficacy for studies included in this analysis up to 22 January 2023 (n = 40 studies selected)

^a^ Fitted least squares regression line.

The Spearman’s correlation coefficient was moderately positive for FI and sample size (0.607), and weakly positive for FQ and sample size (0.023), with considerable variability among both small and large trials (Figure 2). Both FI and FQ were weakly negatively correlated with the percentage LTFU (Spearman correlation coefficient: −0.199 and −0.200, respectively) and showed particularly large variability in trials with low percentage LTFU (Figure 3). Both the FI and FQ showed a weak positive correlation with VE, with Spearman correlation coefficients of 0.138 and 0.161, respectively, and considerable variability among trials with high VE (Figure 4).

Spearman’s correlation coefficient: fragility index vs. sample size 0.607; fragility quotient vs. loss to follow-up 0.023.

Spearman’s correlation coefficient: fragility index vs. loss to follow-up −0.199; fragility quotient vs. loss to follow-up −0.200.

We excluded two outcomes from two studies because they did not report the denominator (number of participants randomised).

Spearman’s correlation coefficient: fragility index vs. vaccine efficacy 0.138; fragility quotient vs. vaccine efficacy 0.161.

Horizontal error bar shows the 95% confidence interval of vaccine efficacy.

**Figure 5:**
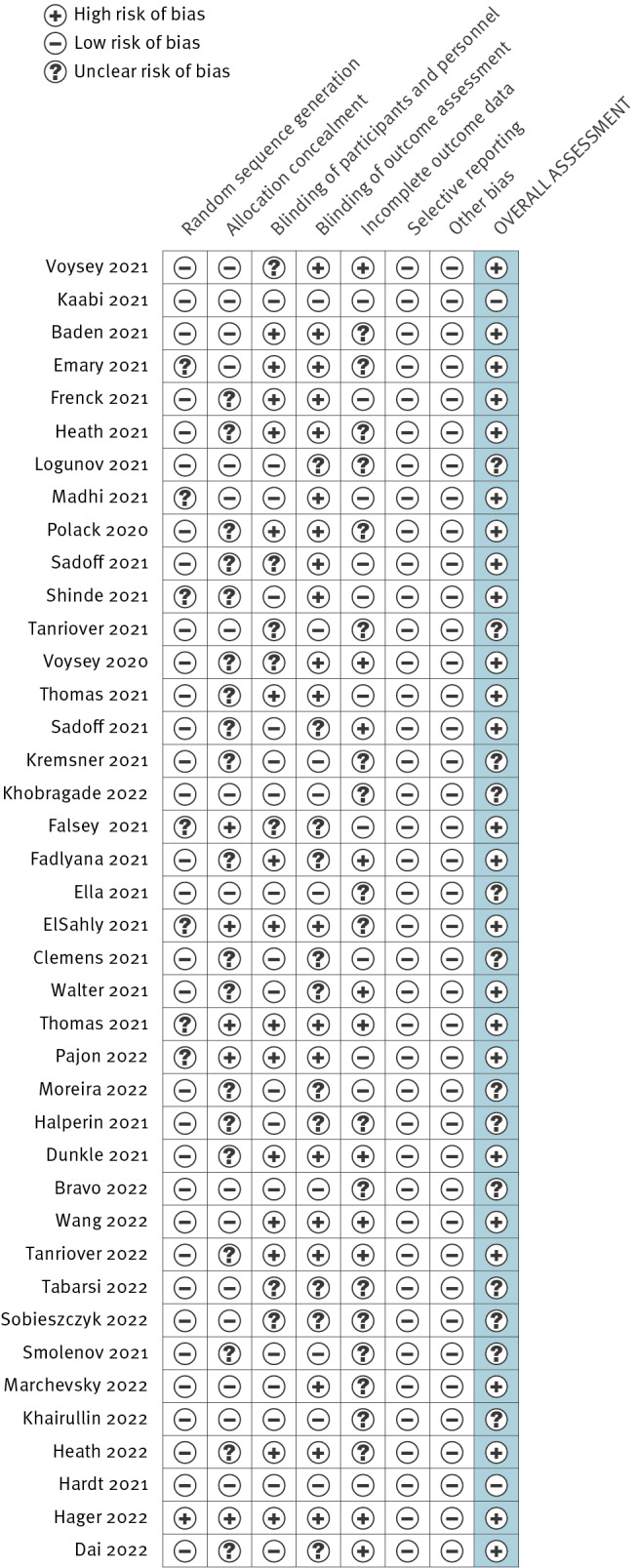
Classification, according to risk of bias, of studies included in this analysis up to 22 January 2023

Based on the RoB2 scale, we assessed two studies to be at low risk of bias and 13 at unclear risk of bias, while most studies (25/40) were classified as at high risk of bias (Figure 5).

More details on each of the studies including their references are available in Table 1.

## Discussion

This comprehensive review assessed of the robustness of COVID-19 vaccine trial results. After reviewing the global evidence and based on an analysis of 909,404 participants across 54 primary outcomes from 40 included trials, we found the median FI and FQ of COVID-19 VE trials to be 62 (IQR: 22–123) and 0.50% (IQR: 0.24–0.92), respectively. Trial robustness increased with sample size and showed substantial variability across a range of other trial characteristics such as blinding type, percentage LTFU and VE.

We found substantially higher robustness levels for COVID-19 VE trials than reported in previous FI reviews of other types of interventions. For example, among the five biggest previous FI reviews [13,18,19,31,32] covering a wide range of interventions and areas, FIs ranged between 8 and 26. Having a large sample size in clinical trials is generally considered a positive aspect, as it increases the power of the study and the ability to detect meaningful differences between groups [33]. However, while it is known that the FI is prone to increase with sample size [17], it is important to note that the median number of study participants in the aforementioned reviews ranged between 682 to 2,548, compared with 13,993 (IQR: 8,534–25,519) in our study. This correlation between sample size and FI can be interpreted as a reflection of the robustness of the trial result. A high FI in a large trial can indicate that results are not easily influenced by a small number of outcome changes, which is a desirable characteristic in clinical trials.

In seven reviews reporting FQ estimates since 2020, the median FQs ranged between 0.7% and 19% [18,20,21,29,34-36]. In our study, the median FQ is 0.50% (IQR: 0.24–0.92). This indicates that, on average, half a percent of study participants would need to have their outcomes changed from an event to a non-event for the trial results to turn from statistically significant to statistically not significant. In other words, the FQ provides a measure of how sensitive trial results are to changes in a small number of participants [37,38]. A low FQ value means that a trial result is relatively robust and not overly sensitive to small changes in the number of outcomes, and vice versa.

It is important to note that most of these abovementioned reviews assessed treatments in non-infectious internal medicine or surgical interventions, and none of them was on vaccines. With regards to COVID-19, one systematic review of FIs reported in trials that assessed the use of corticosteroids to treat acute respiratory distress syndrome in COVID-19 patients found only two of five RCTs, comprising a total of 7,692 patients, to have a FI above 0 (median FI: 6) [38]. Another study attempted to assess FIs in therapeutic interventions and vaccines for COVID-19, and found 47 studies, of which only five were vaccine trials [39]. The median FI reported for these five vaccine studies was 119 (IQR: 61–139), compared with 2.5 (IQR: 1–6) in the 36 included drug trials; FQs were not reported. While this is nearly double the FI compared with our results, a number of methodological issues make a comparison of results difficult. Limitations include that neither study-specific FIs, sample sizes, or risk of bias assessment were presented. Further it is not clear which RCTs were actually included in that review as these were not explicitly listed, which makes that review non-reproducible. The search was restricted to one database only and dates to early August 2021. This might explain the low number of included vaccine studies, as virtually all COVID-19 vaccine trials that informed the rollout of vaccines worldwide were published thereafter. 

While the absence of other similarly large FI reviews limits a meaningful contextualisation of our results, we believe that the overall high median FI found in our study truly reflects substantially higher robustness levels of COVID-19 VE trials compared with other interventions for which FIs have been calculated before. Of note, the ability of the FI to provide sufficient additional value for allowing an enhanced interpretation of trial results beyond what p values can offer has been questioned [40-42]. While the shortfalls of p values are widely accepted, confidence intervals and minimal clinically important differences are sometimes proposed as more informative than the FI [14,43]. In any case, the FI should always be seen as a complementary metric for trial robustness and trustworthiness alongside other established criteria like relative and absolute effect size differences, confidence intervals, p values, clinical significance, and trial design [14].

We use the standard FI methodology developed by Walsh et al. [13] in this study. Modifications to this approach have been proposed recently [44,45], which has initiated an ongoing debate surrounding the advantages and disadvantages of these methods. To date, the method developed by Walsh et al. clearly remains the most widely accepted and frequently used approach for estimating trial fragility. We have therefore made a deliberate decision to use this methodology in our analysis.

Notable strengths of our research include that we closely followed best practice during the conduct and reporting of this study as per Cochrane and PRISMA guidelines wherever applicable; employed comprehensive search statements fitted for the three most relevant databases (Supplementary File 2); covered the three most frequently used, clinically relevant outcomes for COVID-19 vaccine trials; and provided in-depth analyses separately for all eligible primary outcomes of included studies. 

We do, however, acknowledge certain limitations. First, the standard FI and FQ methodology was used in this review can only be applied to studies with randomised allocation of interventions [37]. This led to the exclusion of 121 non-randomised studies during screening (Figure 1). Having said that, decisions by regulatory authorities in most countries about the approval and implementation of COVID-19 vaccines has nearly exclusively been based on randomised studies [41,42]. We therefore consider potential selection bias due to this methodological constraint not to be substantial and thus not to impact the public health relevance of our study. However, in certain specific situations, e.g. when reports emerged in 2021 of rare thrombosis cases associated with the AstraZeneca vaccine, public health advice had to rely initially on anecdotal evidence and on observational studies [46-48]. This was not covered by our review. Second, the FI and FQ as used in this study can only measure robustness in RCTs with dichotomous outcomes and cannot be applied to continuous or time-to-event outcomes due to its underlying principles and methodology. However, since most COVID-19 VE trials have dichotomous outcomes, with only one of our identified studies being excluded for continuous outcome (Figure 1), this did not affect our findings substantially. Third, there is no commonly agreed cut-off value for the FI or FQ to classify an outcome as ‘robust’ or not, making the interpretation of any FI review difficult. Forth, we only searched for trials published in English language. Given the dominance of the English-speaking literature in the global evidence on COVID-19 VE, we do not believe that this caused major problems for our main findings.

## Conclusions

We performed a large, comprehensive systematic review and meta-analysis of COVID-19 vaccine trials. Results from COVID-19 VE trials were substantially more robust than from trials in other areas and other types of interventions. While robustness increased with trial size, both the FI and FQ showed considerable variability across several important trial characteristics. The FI and FQ provide important insights for the interpretation of vaccine trials in particular, and we recommend their reporting alongside established parameters as a complementary indicator for how fragile results are to changes in outcome events. This is specifically relevant for interventions as pivotal as COVID-19 vaccines in a global pandemic.

## Supplementary Data

497000 Supplementary material
