# Supplementary material for: Assessing the robustness of COVID-19 vaccine efficacy trials: systematic review and meta-analysis, January 2023
Source: Euro Surveill. 2023 Jun 1;28(22):2200706. doi: 10.2807/1560-7917.ES.2023.28.22.2200706 (PMC10236928; doi:10.2807/1560-7917.ES.2023.28.22.2200706)
Supplement: Supplementary material [file 2200706_Supplementary_material.pdf]

Supplementary material for the Eurosurveillance research article "Assessing the robustness of COVID-19 vaccine efficacy trials: systematic review and meta-analysis, January 2023"

This supplementary material is hosted by Eurosurveillance as supporting information alongside the article “Assessing the robustness of COVID-19 vaccine efficacy trials: systematic review and meta-analysis, January 2023”, on behalf of the authors, who remain responsible for the accuracy and appropriateness of the content. The same standards for ethics, copyright, attributions and permissions as for the article apply. Supplements are not edited by Eurosurveillance and the journal is not responsible for the maintenance of any links or email addresses provided therein.

Supplement File 1: Example of fragility index calculation

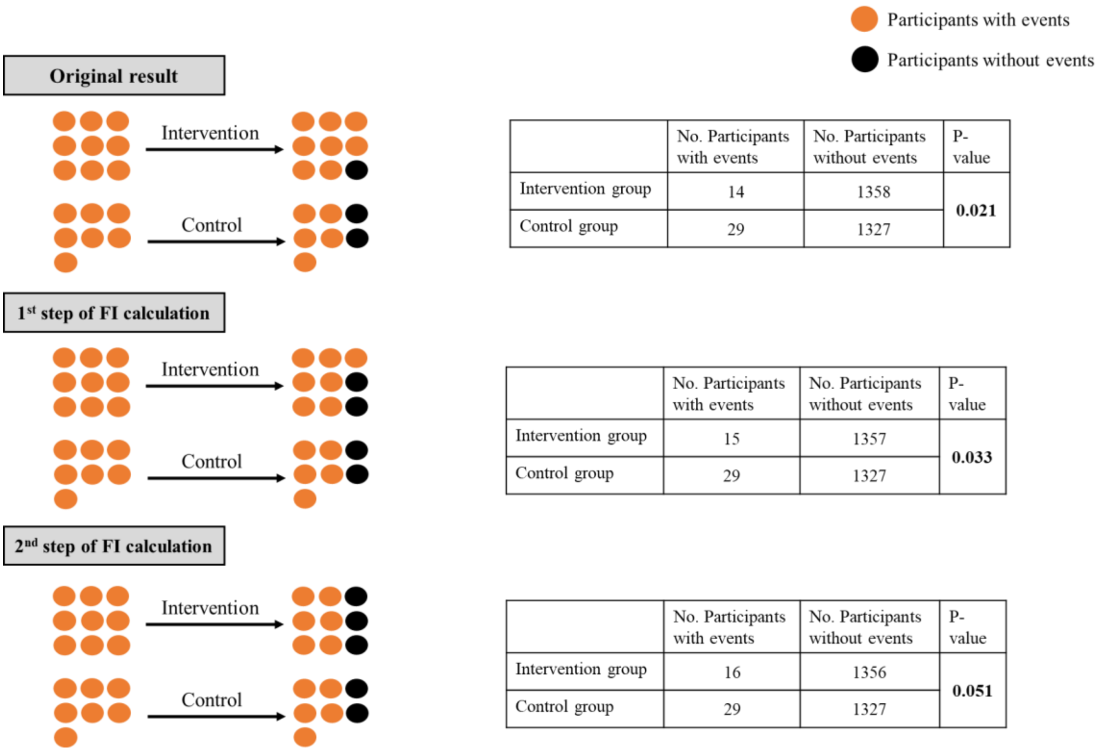

	No. Participants with events	No. Participants without events	P-value
Intervention group	14	1358	**0.021**
Control group	29	1327	

	No. Participants with events	No. Participants without events	P-value
Intervention group	15	1357	**0.033**
Control group	29	1327	

	No. Participants with events	No. Participants without events	P-value
Intervention group	16	1356	**0.051**
Control group	29	1327	

Supplement File 2: Search statements by database

**DATABASE**	
**PubMed**	
	#1: "corona virus"[tw] OR coronavirus[tw] OR coronavirinae[tw] OR coronaviridae[tw] OR betacoronavirus[tw] OR “beta coronavirus” [tw]
	#2: COVID19[tw] OR "COVID 19"[tw] OR COVID2019[tw] OR "COVID 2019"[tw]
	#3: nCoV[tw] OR "CoV 2"[tw] OR CoV2[tw] OR 2019nCoV[tw] OR “2019 nCoV”[tw] OR "severe acute respiratory syndrome coronavirus 2"[tw] OR "SARS CoV 2"[tw] OR sarscov2
	#4: **#1 OR #2 OR #3**
	#5: "COVID-19 Vaccines"[Mesh]
	#6: vaccin*[tw]
	#7: immuniz*[tw]
	#8: inocul*[tw]
	#9: "COVID19 vaccin*"[tw] OR “anti-sars-cov-2 agent”[tw] OR anti-sarscov2 agent[tw] OR sarscov-2 vaccine[tw]
	**#10: #5 OR #6 OR #7 OR #8 OR #9**
	**#11: #4 AND #10**
	#12: Humans[Mesh] OR human*[tw] OR volunteer*[tw] OR participant*[tw] OR subject*[tw] OR people[tw]
	#13: (clinical[tw] AND trial[tiab]) OR clinical trials as topic[Mesh] OR “random allocation”[Mesh] OR clinical trial[tw] OR random*[tw]
	**#14: #12 AND #13**
	**#15: #11 AND #14**
	**Filters applied: English (for language) and Humans (for species)**
**Cochrane Library**	
	#1: MeSH descriptor: [Coronavirus] explode all trees
	#2: MeSH descriptor: [Coronaviridae] explode all trees
	#3: MeSH descriptor: [Coronavirus Infections] explode all trees
	#4: MeSH descriptor: [SARS-CoV-2] explode all trees
	#5: MeSH descriptor: [Betacoronavirus] explode all trees
	#6: coronavir* OR corona virus OR betacoronavir* OR beta coronavirus
	#7: COVID19 OR COVID 19 OR COVID 2019 OR nCoV OR CoV 2 OR CoV2 OR 2019 nCoV OR severe acute respiratory syndrome coronavirus 2 OR SARS CoV 2 OR sarscov2
	#8: **#1 OR #2 OR #3 OR #4 OR #5 OR #6 OR #7**
	#9: MeSH descriptor: [Vaccines] in all MeSH products
	#10: MeSH descriptor: [Immunity] in all MeSH products
	#11: (vaccin*):ti,ab,kw OR (immuniz*):ti,ab,kw OR (inocul*):ti,ab,kw
	#12: **#9 OR #10 OR #11**
	#13: **#8 AND #12**
	#14: MeSH descriptor: [COVID-19 Vaccines] explode all trees
	#15: COVID19 Vaccin* OR COVID 19 Vaccin* OR anti sars cov 2 agent OR anti sarscov2 agent OR sars cov 2 vaccin* OR sarscov2 vaccin*
	#16: **#14 OR #15**
	#17: **#13 OR #16**
	#18: (human*):ti,ab,kw OR (volunteer*):ti,ab,kw OR (participant*):ti,ab,kw OR (subject*):ti,ab,kw OR (people*):ti,ab,kw
	#19: ("clinical trial"):ti,ab,kw OR (random*):ti,ab,kw OR (clinical trial):ti,ab,kw
	#20: **#17 AND #18 AND #19**
**Embase**	

	#1: corona virus.mp. or exp Coronavirinae/
	#2: Coronaviridae/ or exp coronavirus disease 2019/ or exp Severe acute respiratory syndrome coronavirus 2/
	#3: exp Betacoronavirus 1/ or exp Betacoronavirus/
	#4: ('Betacoronavirus' or 'beta coronavirus').mp.
	#5: coronavirus disease 2019.mp. or exp coronavirus disease 2019/
	#6: ('coronavirus disease 2019' or 'coronavirus disease 2019' or 'COVID-19' or COVID19 or sarscov2 or 'COVID-2019' or COVID2019).mp.
	#7: exp severe acute respiratory syndrome/ or exp coronavirus disease 2019/ or exp Severe acute respiratory syndrome coronavirus 2/ or exp SARS coronavirus/
	#8: ('nCoV' or 'SARS-CoV-2' or 2019nCoV or CoV2).mp.
	#9: **#1 OR #2 OR #3 OR #4 OR #5 OR #6 OR #7 OR #8**
	#10: anti-sars-cov-2 agent.mp. or exp anti-SARS-CoV-2 agent/
	#11: 'sars-cov-2 vaccine.mp. or exp SARS-CoV-2 vaccine/
	#12: ("covid19 vaccin*" or "covid 19 vaccin*").mp.
	#13: **#10 OR #11 OR #12**
	#14: **#9 AND #13**
	#15: exp immunization/ OR exp randomization/ OR exp human/
	#16: exp clinical trial/ or exp "controlled clinical trial (topic)"/ or exp controlled clinical trial/ or exp randomized controlled trial/ or exp "randomized controlled trial (topic)"/
	#17: **#14 AND #15 AND #16**
	**Filters applied: English (for language)**

Supplement File 3: Eligibility criteria checklist for full text screening

General Information

**Study ID** (unique assigned number on Covidence)	
**Study title**	
**Notes**	

Study eligibility

Study Characteristics	Eligibility criteria	Eligibility criteria met? Yes	No	Unclear	Location in text or source *(pg/fig/table/other)*
**Type of study**	Randomised controlled trial	☐	☐	☐	
	Frequentist statistical method	☐	☐	☐	
**Participants**	Humans	☐	☐	☐	
**Types of intervention**	Any SARS-CoV-2 vaccines	☐	☐	☐	
**Types of comparison**	Placebo, active control or standard care	☐	☐	☐	
**Types of outcome measures**	Dichotomous outcomes	☐	☐	☐	
	Efficacy of vaccine: reduction the infections	☐	☐	☐	
	Efficacy of vaccine: reduction in morbidity	☐	☐	☐	
	Efficacy of vaccine: reduction in mortality	☐	☐	☐	
	**INCLUDE** ☐			**EXCLUDE** ☐	
**Reason for exclusion**					
**Notes:**					

Supplement File 4: Data extraction form for included studies

General Information

Study ID (unique assigned number on Covidence)	
Study title	
Protocol registration *(yes/no)*	
Journal name	
Publication year	
Journal impact factor *(in the year of publication)*	
Type of funding *(no funding/government/private)*	
Notes	

Methods

	**Descriptions as stated in the article**	**Location in text or source** ***(pg & ¶/fig/table/other)***
Aim of study *(e.g. superiority, non-inferiority, adaptive)*		
RCT design type *(e.g. parallel groups, crossover, factorial, randomized withdrawal)*		
Type of blinding *(e.g. single, double, triple)*		
Number of sites *(single-site, if multi-centre: no of sites)*		
Phase of trials *(II/III, III, IV)*		
Sample size calculation prior to enrolment *(yes/no)*		
Unit of allocation *(by individuals, cluster/ groups)*		
Reported to have obtained ethical approval	☐ Yes ☐ No ☐ Unclear	

Participants

	**Descriptions as stated in the article**	**Location in text or source** ***(pg & ¶/fig/table/other)***
Population description *(from which study participants are drawn, including inclusion, exclusion)*		
Country(ies) *(where participants enrolled)*		
Time *(details time range)*		
Informed consent obtained	☐ Yes ☐ No ☐ Unclear	
Total no. randomised		
Total no. analysed		
Total no. lost to follow-up		
No. lost to follow-up in intervention group		
No. lost to follow-up in control group		
Notes:		

Intervention & Control groups

Copy and paste table for each intervention and comparison group

	**Descriptions as stated in the article**	**Location in text or source** ***(pg & ¶/fig/table/other)***
Name of vaccine<br>*(vaccine brand name)*		
Type of vaccine used *(viral vector, genetic – nucleic acid, inactivated, attenuated, or protein)*		
Name/type of control used *(placebo/active control/standard of care)*		

Outcomes *(Copy and paste table for each efficacy outcome)*

	**Descriptions as stated in the article**	**Location in text or source** ***(pg & ¶/fig/table/other)***
Outcome name		
Types of outcome<br>*(primary/ secondary/ tertiary)*		
Outcome definition *(with diagnostic criteria if relevant)*		
Reported the threshold for determining statistical significance *(yes/no, if yes, specify)*		
Reported statistical significance *(yes/no)*		
Reported p-value *(yes/no, if yes, specify value and the statistical test used)*		
Effect size measure and value *(e.g. RR, OR, IRR, and 95%CI)*		
Type of analysis *(intention to treat/per protocol)*		
Missing data management *(e.g. assumptions made for ITT analysis)*		
Power calculation *(Yes/no, if yes, level of power achieved)*		
Notes:		
**Fragility Index Calculation**		
The number of outcome events in experimental group		
The number of outcome events in control group		
The total number of people in the experimental group included in the analysis		
The total number of people in the control group included in the analysis		
Notes:		

Supplement File 5: List of excluded articles during full text screening

DOI	Reasons
DOI: 10.3390/vaccines10071082	No vaccine efficacy outcomes as primary outcome
DOI: 10.1056/NEJMoa2109522	No vaccine efficacy outcomes as primary outcome
DOI: 10.1038/s41586-021-03681-2	No vaccine efficacy outcomes as primary outcome
DOI: 10.1056/NEJMoa2209367	No vaccine efficacy outcomes as primary outcome
DOI: 10.1056/NEJMoa2028436	No vaccine efficacy outcomes as primary outcome
DOI: 10.1016/j.ijid.2021.10.030	No vaccine efficacy outcomes as primary outcome
DOI: 10.1056/NEJMoa2116414	No vaccine efficacy outcomes as primary outcome
DOI: 10.1056/NEJMc2115597	No vaccine efficacy outcomes as primary outcome
DOI: 10.1038/s41591-020-01179-4	No vaccine efficacy outcomes as primary outcome
DOI: 10.1016/j.annonc.2021.08.1552	No vaccine efficacy outcomes as primary outcome
DOI: 10.1016/j.ebiom.2021.103705	No vaccine efficacy outcomes as primary outcome
DOI: 10.1016/j.ebiom.2021.103705	No vaccine efficacy outcomes as primary outcome
DOI: 10.1136/annrheumdis-2021-221558	No vaccine efficacy outcomes as primary outcome
DOI: 10.1016/s0140-6736(21)01420-3	No vaccine efficacy outcomes as primary outcome
DOI: 10.1093/cid/ciab823	No vaccine efficacy outcomes as primary outcome
DOI: 10.1038/s41467-022-35480-2	No vaccine efficacy outcomes as primary outcome
DOI: 10.1038/s41421-021-00300-2	No vaccine efficacy outcomes as primary outcome
DOI: 10.1101/2022.01.25.22269808	No vaccine efficacy outcomes as primary outcome
DOI: 10.1016/S1473-3099(21)00200-0	No vaccine efficacy outcomes as primary outcome
DOI: 10.1093/cid/ciaa1703	No vaccine efficacy outcomes as primary outcome
DOI: 10.1093/cid/ciab438	No vaccine efficacy outcomes as primary outcome
DOI: 10.1038/s41591-021-01527-y	No vaccine efficacy outcomes as primary outcome
DOI: 10.1016/j.vaccine.2021.02.007	No vaccine efficacy outcomes as primary outcome
DOI: 10.1038/s41591-022-01739-w	No vaccine efficacy outcomes as primary outcome
DOI: 10.1056/NEJMoa2203315	No vaccine efficacy outcomes as primary outcome
DOI: 10.1111/ajt.16701	No vaccine efficacy outcomes as primary outcome
DOI: 10.1016/S1473-3099(21)00070-0	No vaccine efficacy outcomes as primary outcome
DOI: 10.1016/S1473-3099(20)30942-7	No vaccine efficacy outcomes as primary outcome
DOI: 10.1038/s41591-020-01194-5	No vaccine efficacy outcomes as primary outcome
DOI: 10.1186/s40249-021-00924-2	No vaccine efficacy outcomes as primary outcome
DOI: 10.1016/s0140-6736(21)01699-8	No vaccine efficacy outcomes as primary outcome
DOI: 10.1016/s0140-6736(20)31604-4	No vaccine efficacy outcomes as primary outcome
DOI: 10.1038/s41564-022-01262-1	No vaccine efficacy outcomes as primary outcome
DOI: 10.1371/journal.pmed.1003769	No vaccine efficacy outcomes as primary outcome
DOI: 10.1016/s2352-3018(21)00103-x	No vaccine efficacy outcomes as primary outcome
DOI: 10.1101/2022.01.24.22269666	No vaccine efficacy outcomes as primary outcome
DOI: 10.1016/S1473-3099(21)00147-X	No vaccine efficacy outcomes as primary outcome
DOI: 10.7326/acpj202111160-123	No vaccine efficacy outcomes as primary outcome
DOI: 10.1016/j.ajog.2021.03.023	No vaccine efficacy outcomes as primary outcome
DOI: 10.1016/j.eclinm.2021.101010	No vaccine efficacy outcomes as primary outcome
DOI: 10.1016/j.eclinm.2021.101010	No vaccine efficacy outcomes as primary outcome
DOI: 10.1016/S1473-3099(21)00319-4	No vaccine efficacy outcomes as primary outcome
DOI: 10.1016/S1473-3099(21)00319-4	No vaccine efficacy outcomes as primary outcome

DOI: 10.1038/s41467-021-27316-2	No vaccine efficacy outcomes as primary outcome
DOI: 10.1038/s41586-021-04232-5	No vaccine efficacy outcomes as primary outcome
DOI: 10.1182/blood.2021014085	No vaccine efficacy outcomes as primary outcome
DOI: 10.1016/s2213-2600(21)00402-1	No vaccine efficacy outcomes as primary outcome
DOI: 10.3390/vaccines9121375	No vaccine efficacy outcomes as primary outcome
DOI: 10.1016/S2213-2600(21)00557-9	No vaccine efficacy outcomes as primary outcome
DOI: 10.1056/NEJMoa2022483	No vaccine efficacy outcomes as primary outcome
DOI: 10.1056/NEJMoa2026920	No vaccine efficacy outcomes as primary outcome
DOI: 10.1093/cid/ciab1008	No vaccine efficacy outcomes as primary outcome
DOI: 10.1093/infdis/jiac016	No vaccine efficacy outcomes as primary outcome
DOI: 10.1007/s00508-021-01922-y	No vaccine efficacy outcomes as primary outcome
DOI: 10.1016/j.eclinm.2021.101218	No vaccine efficacy outcomes as primary outcome
DOI: 10.1016/j.eclinm.2021.101218	No vaccine efficacy outcomes as primary outcome
DOI: 10.7326/M21-3480	No vaccine efficacy outcomes as primary outcome
DOI: 10.1126/scitranslmed.abj1996	No vaccine efficacy outcomes as primary outcome
DOI: 10.1016/j.ebiom.2021.103810	No vaccine efficacy outcomes as primary outcome
DOI: 10.1007/s40620-021-01076-0	No vaccine efficacy outcomes as primary outcome
DOI: 10.1093/cid/ciac458	No vaccine efficacy outcomes as primary outcome
DOI: 10.1038/s41591-021-01330-9	No vaccine efficacy outcomes as primary outcome
https://DOI.org/10.1080/22221751.2021.1951126	No vaccine efficacy outcomes as primary outcome
DOI: 10.1080/22221751.2021.1937328	No vaccine efficacy outcomes as primary outcome
DOI: 10.1093/infdis/jiab627	No vaccine efficacy outcomes as primary outcome
DOI: 10.1016/s0140-6736(21)01694-9	No vaccine efficacy outcomes as primary outcome
DOI: 10.1016/s0140-6736(20)31866-3	No vaccine efficacy outcomes as primary outcome
DOI: 10.1016/s2352-3018(21)00157-0	No vaccine efficacy outcomes as primary outcome
DOI: 10.1038/s41591-021-01469-5	No vaccine efficacy outcomes as primary outcome
DOI: 10.3389/fimmu.2021.747830	No vaccine efficacy outcomes as primary outcome
DOI: 10.1038/s41392-021-00692-3	No vaccine efficacy outcomes as primary outcome
DOI: 10.1136/bmjopen-2021-056872	No vaccine efficacy outcomes as primary outcome
DOI: 10.1164/rccm.202111-2655LE	No vaccine efficacy outcomes as primary outcome
DOI: 10.1016/j.eclinm.2021.101020	No vaccine efficacy outcomes as primary outcome
DOI: 10.1038/s41586-020-2639-4	No vaccine efficacy outcomes as primary outcome
DOI: 10.1016/s0140-6736(21)02717-3	No vaccine efficacy outcomes as primary outcome
DOI: 10.1016/j.vaccine.2022.03.036	No vaccine efficacy outcomes as primary outcome
DOI: 10.1172/jci.insight.157031	No vaccine efficacy outcomes as primary outcome
DOI: 10.1016/S1470-2045(21)00574-X	No vaccine efficacy outcomes as primary outcome
DOI: 10.1186/s13045-021-01090-6	No vaccine efficacy outcomes as primary outcome
DOI: 10.1016/j.eclinm.2022.101323	No vaccine efficacy outcomes as primary outcome
DOI: 10.1016/j.eclinm.2021.101262	No vaccine efficacy outcomes as primary outcome
DOI: 10.1101/2022.02.08.22270676	No vaccine efficacy outcomes as primary outcome
DOI: 10.1016/j.vaccine.2021.04.006	No vaccine efficacy outcomes as primary outcome
DOI: 10.1016/s0140-6736(20)32466-1	No vaccine efficacy outcomes as primary outcome
DOI: 10.1001/jamainternmed.2021.7372	No vaccine efficacy outcomes as primary outcome
DOI: 10.1016/s0140-6736(21)00241-5	No vaccine efficacy outcomes as primary outcome
DOI: 10.15789/2220-7619-ASB-1699	No vaccine efficacy outcomes as primary outcome
DOI: 10.1056/NEJMoa2116747	No vaccine efficacy outcomes as primary outcome

DOI: 10.1056/NEJMoa2034201	No vaccine efficacy outcomes as primary outcome
DOI: 10.1038/s41586-020-2814-7	No vaccine efficacy outcomes as primary outcome
DOI: 10.1038/s41586-021-03653-6	No vaccine efficacy outcomes as primary outcome
DOI: 10.1111/jgs.17153	No vaccine efficacy outcomes as primary outcome
DOI: 10.1016/j.ccell.2021.11.006	No vaccine efficacy outcomes as primary outcome
DOI: 10.1093/rheumatology/keab773	No vaccine efficacy outcomes as primary outcome
DOI: 10.1038/s41591-021-01542-z	No vaccine efficacy outcomes as primary outcome
DOI: 10.1097/cm9.0000000000001702	No vaccine efficacy outcomes as primary outcome
DOI: 10.3390/cancers13143573	No vaccine efficacy outcomes as primary outcome
DOI: 10.1093/cid/ciac169	No vaccine efficacy outcomes as primary outcome
DOI: 10.1016/S1473-3099(21)00764-7	No vaccine efficacy outcomes as primary outcome
DOI: 10.1001/jama.2021.3645	No vaccine efficacy outcomes as primary outcome
DOI: 10.1016/s0140-6736(21)02718-5	No vaccine efficacy outcomes as primary outcome
DOI: 10.1200/JCO.2021.39.15_suppl.6510	No vaccine efficacy outcomes as primary outcome
DOI: 10.1371/journal.pmed.1004024	No vaccine efficacy outcomes as primary outcome
DOI: 10.1016/j.ebiom.2021.103811	No vaccine efficacy outcomes as primary outcome
DOI: 10.1056/NEJMoa2027906	No vaccine efficacy outcomes as primary outcome
DOI: 10.1172/JCI157707	No vaccine efficacy outcomes as primary outcome
DOI: 10.1038/s41591-021-01370-1	No vaccine efficacy outcomes as primary outcome
DOI: 10.1172/jci149335	No vaccine efficacy outcomes as primary outcome
DOI: 10.1016/S1473-3099(21)00396-0	No vaccine efficacy outcomes as primary outcome
DOI: 10.1016/S1473-3099(20)30987-7	No vaccine efficacy outcomes as primary outcome
DOI: 10.1001/jama.2020.15543	No vaccine efficacy outcomes as primary outcome
DOI: 10.1016/S1473-3099(20)30831-8	No vaccine efficacy outcomes as primary outcome
DOI: 10.1016/S1473-3099(20)30831-8	No vaccine efficacy outcomes as primary outcome
DOI: 10.1016/S1473-3099(20)30831-8	No vaccine efficacy outcomes as primary outcome
DOI: 10.1016/s1473-3099(21)00462-x	No vaccine efficacy outcomes as primary outcome
DOI: 10.1016/S1473-3099(21)00127-4	No vaccine efficacy outcomes as primary outcome
DOI: 10.1016/j.eclinm.2021.101078	No vaccine efficacy outcomes as primary outcome
DOI: 10.1016/S1473-3099(21)00681-2	No vaccine efficacy outcomes as primary outcome
DOI: 10.1080/22221751.2021.1951126	No vaccine efficacy outcomes as primary outcome
DOI: 10.3389/fimmu.2022.841868	Not SARS-CoV-2 vaccine as intervention
DOI: 10.1016/j.xcrm.2022.100728	Not SARS-CoV-2 vaccine as intervention
DOI: 10.1038/s41541-021-00394-5	Not using RCT study design
DOI: 10.1002/jmv.27214	Not using RCT study design
DOI: 10.1016/s2468-1253(21)00024-8	Not using RCT study design
DOI: 10.1001/jama.2021.7152	Not using RCT study design
DOI: 10.1016/j.vaccine.2021.06.054	Not using RCT study design
DOI: 10.1172/JCI154834	Not using RCT study design
DOI: 10.1016/s0140-6736(22)00007-1	Not using RCT study design
DOI: 10.3324/haematol.2021.279196	Not using RCT study design
DOI: 10.1016/j.puhe.2021.01.011	Not using RCT study design
DOI: 10.1093/infdis/jiab262	Not using RCT study design
DOI: 10.5603/GP.a2021.0241	Not using RCT study design
https://DOI.org/10.3390/vaccines9040341	Not using RCT study design
DOI: 10.1016/j.addr.2021.01.014	Not using RCT study design

https://DOI.org/10.1038/s41591-021-01446-y	Not using RCT study design
DOI: 10.1186/s40249-021-00878-5	Not using RCT study design
DOI: 10.3390/vaccines9060582	Not using RCT study design
DOI: 10.1001/jamanetworkopen.2021.15985	Not using RCT study design
DOI: 10.3389/fimmu.2022.1032411	Not using RCT study design
DOI: 10.1056/NEJMoa2101765	Not using RCT study design
https://DOI.org/10.1016/j.therap.2021.05.004	Not using RCT study design
DOI: 10.1007/s10389-022-01707-1	Not using RCT study design
DOI: 10.3390/vaccines10010095	Not using RCT study design
https://DOI.org/10.1016/j.cmi.2021.05.004	Not using RCT study design
DOI: 10.1080/22221751.2021.1953403	Not using RCT study design
DOI: 10.1016/s0140-6736(22)00152-0	Not using RCT study design
DOI: 10.1038/s41571-022-00610-8	Not using RCT study design
DOI: 10.1016/j.cmi.2021.10.005	Not using RCT study design
DOI: 10.1136/annrheumdis-2021-220647	Not using RCT study design
DOI: 10.1016/j.cmi.2021.09.036	Not using RCT study design
DOI: 10.1016/j.clim.2021.108786	Not using RCT study design
DOI: 10.3390/v13030422	Not using RCT study design
DOI: 10.14309/01.ajg.0000777772.10234.84	Not using RCT study design
DOI: 10.1053/j.gastro.2021.06.014	Not using RCT study design
DOI: 10.1056/NEJMoa2201300	Not using RCT study design
DOI: 10.1016/S1473-3099(22)00506-0	Not using RCT study design
DOI: 10.1016/j.healun.2021.05.004	Not using RCT study design
DOI: 10.1056/NEJMc2107809	Not using RCT study design
DOI: 10.1016/S1473-3099(22)00416-9	Not using RCT study design
DOI: 10.2807/1560-7917.ES.2021.26.6.2100096	Not using RCT study design
DOI: 10.1056/NEJMoa2107715	Not using RCT study design
DOI: 10.1016/j.vaccine.2021.12.044. Epub 2021 Dec 24	Not using RCT study design
DOI: 10.1002/jmv.26996	Not using RCT study design
DOI: 10.1016/j.ijid.2021.04.047	Not using RCT study design
DOI: 10.7499/j.issn.1008-8830.2101133	Not using RCT study design
DOI: 10.1053/j.gastro.2021.05.044	Not using RCT study design
DOI: 10.1208/s12249-021-02058-y	Not using RCT study design
DOI: 10.1093/ndt/gfab186	Not using RCT study design
DOI: 10.1126/sciadv.abe8065	Not using RCT study design
DOI: 10.1371/journal.pone.0260733	Not using RCT study design
DOI: 10.1007/s10787-021-00839-2	Not using RCT study design
DOI: 10.1002/jmv.27568	Not using RCT study design
DOI: 10.1080/14760584.2021.1949293	Not using RCT study design
DOI: 10.1016/j.jval.2021.04.565	Not using RCT study design
DOI: 10.1136/bmj-2021-068632	Not using RCT study design
DOI: 10.1172/jci149154	Not using RCT study design
DOI: 10.5582/ddt.2021.01058	Not using RCT study design
DOI: 10.1093/cid/ciab630	Not using RCT study design
DOI: 10.1093/cid/ciab226	Not using RCT study design
Antivir Ther. 2007;12(7):1107-13. PMID: 18018769	Not using RCT study design

DOI: 10.1002/jmv.27203	Not using RCT study design
DOI:https://DOI.org/10.1016/S2352-3026(21)00169-1	Not using RCT study design
DOI: 10.2807/1560-7917.ES.2021.26.21.2100438	Not using RCT study design
DOI: 10.1097/TP.0000000000004036	Not using RCT study design
DOI: 10.1182/blood.2021013768	Not using RCT study design
DOI: 10.1016/S1470-2045(21)00213-8	Not using RCT study design
DOI: 10.1111/fcp.12715	Not using RCT study design
DOI: 10.1056/NEJMoa2200674	Not using RCT study design
DOI: 10.1016/j.intimp.2021.107763	Not using RCT study design
DOI: 10.15585/mmwr.mm7113e2	Not using RCT study design
DOI: 10.1001/jamanetworkopen.2022.0935	Not using RCT study design
DOI: 10.15585/mmwr.mm7042e1	Not using RCT study design
DOI: 10.1186/s13063-020-04775-4	Not using RCT study design
DOI: 10.1097/CM9.0000000000001573	Not using RCT study design
DOI: 10.1097/CM9.0000000000001573	Not using RCT study design
DOI: 10.1097/CM9.0000000000001573	Not using RCT study design
DOI: 10.1016/j.cmi.2021.06.043	Not using RCT study design
DOI: 10.1016/j.medj.2021.06.007	Not using RCT study design
DOI: 10.3390/vaccines9050467	Not using RCT study design
DOI: https://DOI.org/10.1002/ygh2.473	Not using RCT study design
DOI: 10.1038/s41591-021-01410-w	Not using RCT study design
DOI: 10.1038/s41591-021-01410-w	Not using RCT study design
DOI: 10.1111/bjh.17982	Not using RCT study design
DOI: 10.18176/jiaci.0683	Not using RCT study design
DOI: 10.1136/bmj.n1087	Not using RCT study design
DOI: 10.7326/ACPJ202107200-075	Not using RCT study design
DOI: 10.7326/ACPJ202111160-124	Not using RCT study design
DOI: 10.1038/s41586-020-2814-7	Not using RCT study design
DOI: 10.3126/nje.v11i1.36163	Not using RCT study design
DOI: 10.3126/nje.v11i1.36163	Not using RCT study design
DOI: 10.1016/j.jceh.2021.06.013	Not using RCT study design
DOI: 10.1681/ASN.2021060778	Not using RCT study design
DOI: 10.1016/S0140-6736(21)00790-X	Not using RCT study design
DOI: https://DOI.org/10.1182/blood-2021-153122	Not using RCT study design
DOI: 10.1016/j.cmi.2022.09.001	Not using RCT study design
DOI: 10.1093/cid/ciab229	Not using RCT study design
DOI: 10.1016/j.vaccine.2021.12.046	Not using RCT study design
DOI: 10.1016/j.annonc.2021.08.1551	Not using RCT study design
DOI: 10.1016/j.annonc.2021.08.1551	Not using RCT study design
DOI: 10.15585/mmwr.mm7013e3	Not using RCT study design
DOI: 10.1016/s2213-2600(21)00409-4	Not using RCT study design
DOI: 10.1016/j.lana.2022.100423	Not using RCT study design
DOI: 10.1186/s13045-021-01205-z	Not using RCT study design
DOI: 10.3390/v13010054	Not using RCT study design
DOI: https://DOI.org/10.22159/ijap.2021v13i4.41270	Not using RCT study design

**DOI:** https://DOI.org/10.1101/2021.11.30.21267102	Not using RCT study design
DOI: 10.1016/S0140-6736(21)00677-2	Not using RCT study design
DOI: 10.1158/2159-8290.Cd-21-1072	Not using RCT study design
DOI: 10.1016/j.jhep.2021.04.026	Not using RCT study design
DOI: 10.1056/NEJMoa2113017	Not using RCT study design
DOI: 10.1056/NEJMc2032195	Not using RCT study design
DOI: 10.1038/s41467-022-29159-x	Not using RCT study design
DOI: 10.7499/j.issn.1008-8830.2101133	Not using RCT study design
DOI: 10.3390/ph14050406	Not using RCT study design
DOI: 10.1056/NEJMoa2110345	Not using RCT study design
DOI: 10.1080/14760584.2021.1925112	Not using RCT study design
DOI: 10.1016/s1473-3099(20)30843-4	Not using RCT study design
DOI: 10.1016/s1473-3099(20)30843-4	Not using RCT study design
DOI: 10.1016/s1473-3099(20)30843-4	Not using RCT study design
DOI: 10.1016/s0140-6736(20)31605-6	Not using RCT study design
DOI: 10.1016/s0140-6736(20)31208-3	Not using RCT study design
DOI: 10.1016/s0140-6736(20)31208-3	Not using RCT study design
DOI: 10.1182/blood.2021011568	Outcome event not dichotomous
